# Treatment of 503 cattle with traumatic reticuloperitonitis

**DOI:** 10.1186/s13028-018-0410-8

**Published:** 2018-09-17

**Authors:** Ueli Braun, Sonja Warislohner, Christian Gerspach, Stefanie Ohlerth, Karl Nuss

**Affiliations:** 1 Department of Farm Animals, Vetsuisse Faculty, University of Zurich, Zurich, Switzerland; 2 Clinic of Diagnostic Imaging, Vetsuisse Faculty, University of Zurich, Zurich, Switzerland

**Keywords:** Cattle, Traumatic reticuloperitonitis, Treatment, Rumenotomy

## Abstract

**Background:**

The treatment of traumatic reticuloperitonitis (TRP) in cattle has a long and impressive history that goes back more than 100 years. This study describes treatment for TRP in 503 cattle. Initial treatment was based on radiographic findings; cattle with a foreign body attached to a magnet were treated conservatively using antibiotics, anti-inflammatory drugs and intravenous fluids. Cattle with a foreign body lying on the ventral aspect of the reticulum or penetrating or perforating the reticulum received a magnet in addition to medical treatment. Cattle were radiographed again the next day. When the foreign body was completely attached to the magnet, medical treatment was continued. When the foreign body was not attached or still penetrated/perforated the reticulum, a rumenotomy was carried out.

**Results:**

Of the 503 cattle, 232 were treated conservatively, 206 underwent surgery, 61 were slaughtered or euthanased and four were treated after discharge at home with a magnet and antibiotics. Surgical treatment was significantly more successful than conservative treatment; 90% of 206 operated and 82% of 232 medically-treated cattle were discharged.

**Conclusions:**

For practical purposes, cattle suspected of having traumatic reticuloperitonitis should initially be treated with a magnet and antibiotics and re-evaluated, ideally radiographically, when response to treatment does not occur within 3 or 4 days. Surgery is limited to cases in which the foreign body fails to completely attach to the magnet.

## Background

The treatment of traumatic reticuloperitonitis (TRP) in cattle has a long and impressive history that goes back more than 100 years and has been documented in detail [1]. In the pre-antibiotic era, cows suspected of having TRP were fasted 2 or 3 days and positioned on an incline to elevate the front end for 1–2 weeks [2]. In addition, cows were treated with antibacterial drugs and laxatives. The first account of removal of a foreign body by means of rumenotomy was in the nineteenth century. Attempts were made to remove metallic foreign bodies using a magnetic probe that was introduced orally [3–5]. Even though this method was sometimes successful in removing superficial and non-perforating foreign bodies, penetrating or perforating foreign bodies that caused disease were rarely retrieved [6]. Currently, treatment of TRP may be conservative or surgical [2, 7, 8]. Conservative treatment consists of oral administration of a regular magnet or a magnet with a plastic cage combined with the administration of antibiotics. The success of treatment with a magnet is well documented [9–12] and in one study could be confirmed radiographically [13]. However, not all magnets fall directly into the reticulum; some fall into the anterior blind sac or possibly elsewhere in the rumen. Attempts to guide a magnet into the reticulum on a piece of string were unsuccessful in one study [14] as were attempts to direct the course of an orally-administered magnet using a second magnet externally. Another study showed that fasting the cow for 1 day, or lowering the position of the forelimbs during application increases the likelihood of a magnet falling into the reticulum [15]. Subcutaneous administration of 40–60 mg atropine 10 min before administration of a magnet was yet another attempt to guide it directly into the reticulum [12, 14]. A compass was used to monitor the final position of the magnet. Follow-up studies showed that the administration of atropine does not affect the placement of a magnet [16–19] even though atropine delays the passage of the magnet through the oesophagus and causes reticular atony [20]. Likewise, lowering the position of the forefeet and pre-treatment with scopolamine or xylazine had no effect [18, 19]. According to current opinion, it is not possible to influence reticular placement of a magnet by means of external manipulations or drugs. Acute and uncomplicated TRP is treated with a magnet combined with antibiotics for several days [2] using penicillin or broad-spectrum antibiotics including tetracyclines or trimethoprim sulfonamide combinations [8]. However, the choice of antibiotics is arbitrary and not based on scientific studies. If no improvement occurs within 2–4 days, surgical treatment to remove the foreign body or euthanasia should be considered [2]. There are two main surgical techniques for rumenotomy [2]; the first involves permanently suturing the rumen to the peritoneum and transverse fascia, which allows extraperitoneal access to the rumen as well as extraperitoneal healing of the rumenotomy incision, and the second is the Weingarth’s ring rumenotomy, in which the rumen is closed after exploration and returned to its normal abdominal position [2]. There have been other studies of laparorumenotomy techniques [21–23], one of which determined that skin suture fixation was superior to the Weingarth’s ring technique [22]. Of 38 cattle that underwent rumenotomy, 34% were still in the herd, 37% had been removed from the herd and 29% had died or been euthanased at follow-up 5 months to 5 years after surgery [24]. The goal of the present study was to describe the treatment and course of the disease in 503 cattle diagnosed with TRP based on the results of clinical, ultrasonographic and radiographic findings.

## Methods

### Animals

A total of 503 cattle treated for TRP at the Department of Farm Animals of the Vetsuisse Faculty, University of Zurich, between January 1, 2001 and December 31, 2014 were analysed [25]. Radiography was used to diagnose TRP in 484 cattle, and laparoruminotomy (206) and postmortem examination (61) were additional diagnostic aids. There were 496 females and 7 males, which ranged in age from 1.0 to 14.9 years (median, 4.1 years) with 97% of the cattle being more than 2 years of age. Breeds included Swiss Braunvieh (208), Holstein–Friesian (155), Simmental (124), Jersey (3), Eringer (1), Hinterwälder (1) and crossbred cattle (11). The month when the animal became ill and the history were recorded in all cattle. The clinical, laboratory, ultrasonographic and radiographic findings obtained on admission to the clinic were published separately [26, 27]. Based on the ultrasonographic findings, 33 cattle with severe and extensive or generalised peritonitis were euthanased.

### Overview of the treatment of TRP

**Fig. 1:**
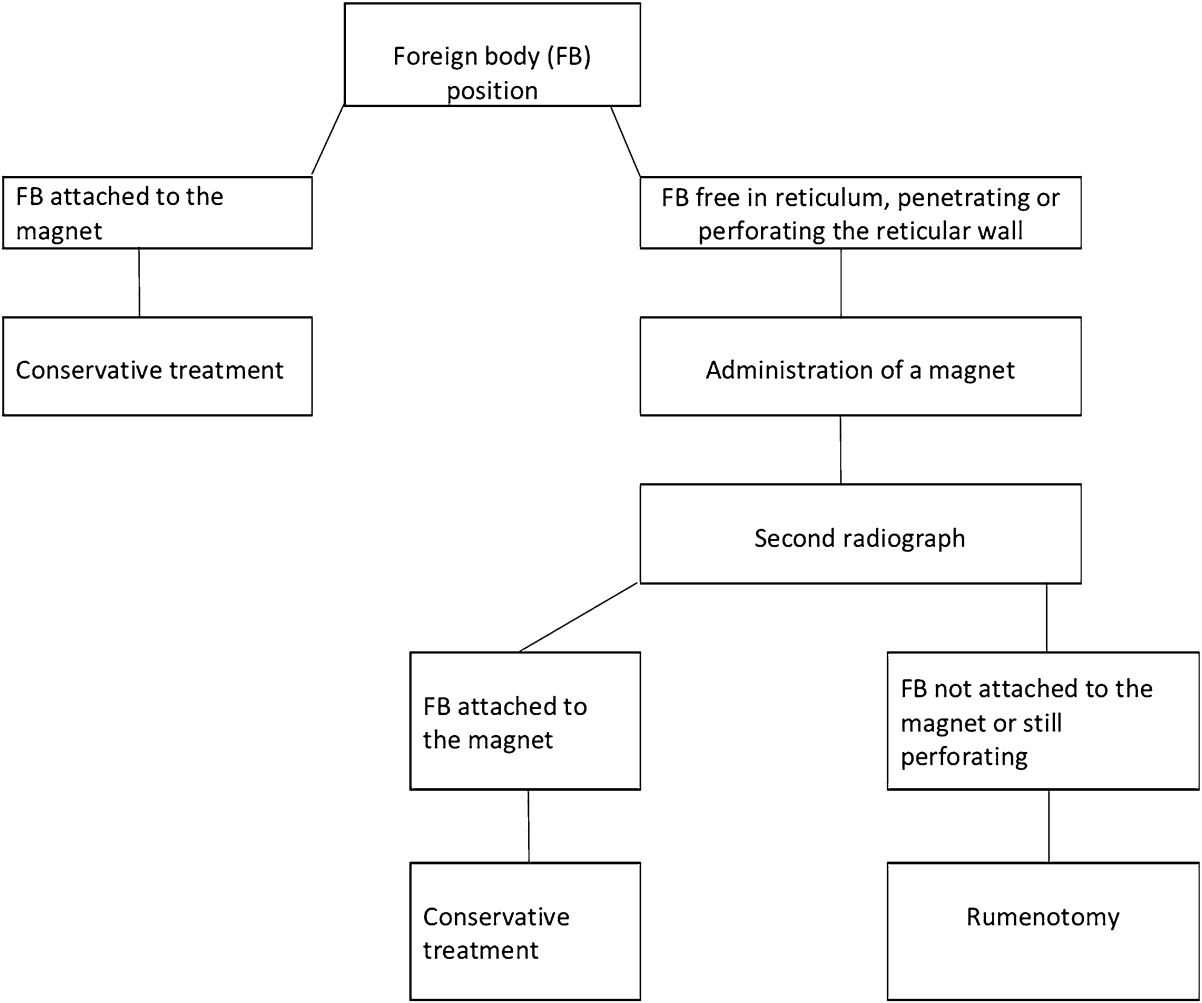
Treatment flowchart. Treatment flowchart for 503 cattle with traumatic reticuloperitonitis

Initial treatment was selected on the basis of radiographic findings at the time of admission (Fig. 1). Cattle with a foreign body attached to a magnet were treated conservatively, and cattle with radiographic evidence of a foreign body (non-penetrating, penetrating or perforating) were treated with antibiotics and a magnet with a plastic cage (Bovivet Magnet, Kruuse, Denmark). The magnet was administered using an appropriate balling gun without premedication of the animal. Radiographic examination was repeated on the following day [28–30] and the results were used to guide subsequent treatment. Conservative treatment was continued when the foreign body was completely attached to the magnet and another radiograph was made. In cases where the foreign body was not in contact with the magnet or was still penetrating or perforating the reticulum, surgery was carried out.

### Conservative treatment

Conservative treatment included amoxicillin (7 mg/kg body weight, Clamoxyl^®^, Zoetis Switzerland) or penicillin G procaine (12,000 IU/kg body weight, Procacillin^®^, MSD Animal Health) given intramuscularly for 1–13 days, in most cases for 7 (n = 42) to 8 (n = 30) days (median 6 days). Seventy-nine cattle received a daily injection of a non-steroidal anti-inflammatory drug (flunixin meglumine, 1 mg/kg, Flunixine^®^, Biokema, Crissier, or ketoprofen, 3 mg/kg, Rifen^®^, Streuli Pharma) or a pyrazolone preparation (metamizole, 35 mg/kg, Vetalgin^®^, MSD Animal Health). All cattle received 10 l of a solution containing 50 g glucose and 9 g sodium chloride per litre daily for 3 days administered as a slow intravenous drip via an indwelling jugular vein catheter (Abbocath-T 14 g, length 14 cm, Abbott AG, Baar). Cows with hypocalcaemia (calcium < 2.0 mmol/L), hypophosphataemia (inorganic phosphorus < 1.0 mmol/L) or hypomagnesaemia (magnesium < 0.7 mmol/L) were treated orally with monocalcium phosphate, sodium dihydrogen phosphate and/or magnesium oxide. Clinical examinations were carried out daily.

### Surgical treatment

Cattle were fasted for 24 h before surgery to reduce rumen fill. After paravertebral anaesthesia of the last thoracic and the first two lumbar nerves on the left, a 25–30 cm incision was made in the abdominal wall parallel to the contour of the last rib. A perforating foreign body was removed from the reticulum during exploration of the abdomen in three cows. In all other cases, foreign bodies were removed via rumenotomy. Adhesive drapes and surgical drapes moistened with sterile saline solution were used to prevent contamination of the surgical field and abdominal cavity. Twenty-one cattle had perireticular abscesses adherent to the reticulum, which were drained after aspiration of the contents and ultrasound-guided incision from within the reticulum. A new cage magnet was placed into the reticulum, and 4–5 L of rumen liquor from a healthy donor cow supplemented with hay chaff was added to the rumen contents before the rumen was closed using a two-layer Cushing suture pattern (PDS 2^®^, Ethicon; since 2011 MonoPlus^®^, B. Braun). The suture line was lavaged with sterile saline-iodine solution, and the abdominal wall was closed routinely in four layers. The cattle were fasted for 24 h after surgery and treated with antibiotics for 1–19 days (median, 8 days), and 201 cattle received flunixin meglumine daily for 3 days, both at the doses outlined for conservative treatment. Two animals were euthanased during surgery because of severe inflammatory changes. Intravenous treatment with glucose and electrolytes was the same as that used for conservative treatment.

### Statistical analysis

The program IBM SPSS Statistics 22.0 was used for analysis. Means ± standard deviations were calculated for normal data and medians were calculated for non-normal data. For normal data, differences between two groups were analysed using a t-test. The Mann–Whitney U-test was used to analyse differences of non-normal data between two groups, and the Chi square test was used to analyse differences of nominal data between two groups. Differences were considered significant at P < 0.05.

## Results

**Fig. 2:**
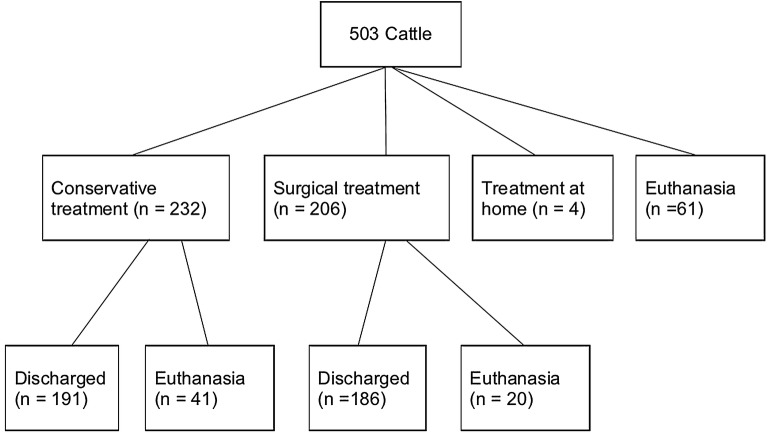
Treatment choices in 503 cattle. Treatment choices in 503 cattle with traumatic reticuloperitonitis

Of the 503 cattle, 232 received conservative treatment, 206 were operated, 61 were slaughtered or euthanased and four were discharged and treated conservatively at home at the request of the owner (Fig. 2). Treatment at home consisted of administration of a magnet as well as antibiotics, such as amoxicillin or penicillin, for several days.

### Supplemental radiographs

A second radiograph was taken in 177 cattle one the day after the administration of a magnet. A third radiograph was taken in 37 cattle 2 days, and a fourth in eight cattle 3 days after the administration of a magnet. Radiographs taken in 177 cattle after administration of a magnet showed the magnet in the reticulum in 146 (82.5%) and in the anterior blind sac of the rumen in 16 (9%). A magnet was not seen on 15 (8.5%) radiographs, most likely because it was in the rumen (Table 1).

**Table 1:** Position and efficacy of a magnet administered orally in 177 cattle with traumatic reticuloperitonitis

Variable	Outcome	n	%
Position of magnet (n = 177)	Reticulum	146	82.5
	Anterior blind sac of rumen	16	9.0
	Not visible	15	8.5
Efficacy of magnet in reticulum (n = 177)	Magnet not in reticulum	31	17.5
	Entire foreign body attached to magnet	94	53.0
	Parts of foreign body protruding from magnet	10	6.0
	No contact between magnet and foreign body	35	20.0
	Not all foreign bodies attached to magnet	6	3.0
	Foreign body not seen on first radiograph	1	0.5

In 94 (53%) cattle with a magnet in the reticulum, the foreign body was attached to the magnet in its entirety and treatment was considered successful. In 10 (6%) cattle, a part of the foreign body was attached to the magnet but one end was protruding and possibly still penetrating the reticulum, and in 35 (20%) cases, the foreign body had maintained its position and had no contact with the magnet. In 6 (3%) cattle with multiple foreign bodies, at least one had remained penetrating or perforating and was not in contact with the magnet. In one (0.5%) animal, the second radiograph showed a foreign body that was missed on the initial radiograph.

The efficacy of treatment with a magnet was significantly associated with the position of the foreign body (P < 0.01, χ^2^ = 11.6). The best efficacy was achieved when the foreign body had an upright position on the ventral aspect of the reticulum; this occurred in 32 cattle and treatment was successful in 24 (75%) (Table 2). Of 56 foreign bodies lying on the ventral aspect of the reticulum, 32 (57%) were attached to the magnet. Of 36 foreign bodies that did not contact the ventral aspect of the reticulum, 16 (44%) were attached to the magnet. Perforation of the reticulum by a foreign body was confirmed radiographically in 22 cattle and in 32% of these, the foreign body was attached to the magnet and completely removed from the reticular wall.

**Table 2:** Efficacy of a magnet in the reticulum dependent on the position of the foreign body in 146 cattle with traumatic reticuloperitonitis

Position of foreign body	Efficacy of magnet	
	n	%
Upright on ventral aspect of reticulum at an angle of > 30 degrees (n = 32)	24	75
Flat on ventral aspect of reticulum (n = 56)	32	57
No contact with ventral aspect of reticulum (n = 36)	16	44
Perforating reticular wall (n = 22)	7	32

χ^2^ = 11.6

P < 0.01

In 37 cattle in which a penetrating foreign body was seen on the second radiograph, a third radiograph showed the foreign body completely attached to the magnet in 24 cattle and without attachment in 13. Five of the latter underwent rumenotomy and eight underwent radiography for the fourth time. In four cattle, the foreign body was attached to the magnet and in four it was not; the latter underwent rumenotomy.

### Efficacy of conservative treatment

Of 232 cattle, 191 (82%) were treated successfully and discharged (Fig. 2) and 41 did not respond to treatment and were euthanased. Nonresponse to treatment was defined as complete or partial anorexia, absent or reduced rumination or pyrexia for several days after the start of treatment. The general health condition and the appetite normalised after 2–14 days (median 2 days) in all but 5 of the cattle that were discharged. The rectal temperature decreased from 39.0 ± 0.61 °C on admission to 38.7 ± 0.35 °C 8 days after the start of treatment, and successfully treated cattle were discharged after 2–18 days (median 8 days).

### Efficacy of surgical treatment

Of 206 cattle that had surgical treatment, 195 underwent standing laparoruminotomy using the Weingarth’s ring, 1 had a laparotomy in dorsal recumbency under general anaesthesia and 10 underwent draining of a reticular abscess through a transcutaneous incision without laparotomy. Surgical treatment was successful in 186 (90%) of 206 cases (Fig. 2), and a foreign body was removed in 184. A return to satisfactory general health and appetite occurred one to 20 days (median 3 days) after surgery. The rectal temperature decreased from 39.0 ± 0.61 °C on admission to 38.8 ± 0.43 °C 8 days after surgery, and successfully treated cattle were discharged after 5–39 days (median 11 days). Twenty animals did not respond to treatment and were euthanased.

### Comparison of conservative and surgical treatment

The efficacy of surgical treatment was significantly greater than that of conservative treatment (90 vs. 82%, P < 0.05), but recovery with regard to general health, appetite and rectal temperature after the start of treatment did not differ between the two groups. Hospitalisation was significantly shorter in conservatively treated cattle (8 vs. 11 days, P < 0.01).

### Type of foreign bodies

The type of 299 foreign bodies retrieved from 271 cattle was documented. There were 141 pieces of fencing wire, 121 nails, 13 screws, 8 flat pieces of metal, 5 pieces of barbed wire, 4 arms from eye glasses, 2 staples, 1 hair clip, 1 piece of aluminium, 1 fence insulator, 1 buckle and 1 set of nail clippers. The foreign bodies were from 1.5 to 18 cm in length (median 6 cm), and 273 were ferromagnetic.

## Discussion

Traumatic reticuloperitonitis may be treated conservatively or surgically [2, 7, 8]. In the present study, the choice of treatment was based on the radiographic findings of the reticulum: cattle with a foreign body attached to a magnet were treated conservatively, and cattle without a magnet were given one orally and radiographed again the next day. Thirty-seven cattle were radiographed a third time and eight cattle a fourth time because the efficacy of the magnet was poor and the owners declined rumenotomy. From a clinical standpoint, it was important to learn that attachment of the foreign body to the magnet was not seen radiographically until the third radiograph in 24 of 37 cattle and the fourth radiograph in four of eight cattle. Thus, the clinical recommendation to wait several days after magnet administration before opting for surgical treatment or euthanasia is reasonable because attachment of a foreign body to a magnet may require a few days. Eating and rumination variables of cows with TRP were shown to normalise rapidly within 3–4 days after successful treatment with a magnet [31]. On the other hand, unnecessary delay of surgery in cattle with a perforating foreign body carries the risk of complications such as traumatic pericarditis or abscess of the liver or spleen.

The efficacy of the magnet in the present study was similar to that of a previous study of 100 cows with TRP [18, 30], in which 85% of magnets were in the reticulum (compared with in 82.5% in the present study), 9% were in the anterior blind sac of the rumen (same as present study) and 6% were not seen on radiographs (compared with 8.5%), most likely because they were in the rumen. Thus, 15–20% of magnets administered orally are likely to end up in the anterior blind sac or other parts of the rumen rather than in the reticulum. This is irrelevant when a magnet is administered prophylactically because most magnets that fall into the rumen are moved into the reticulum within one to 3 days by reticulo-ruminal contractions [2]. However, this process may be delayed or does not occur in sick cows with reduced ruminal contractility, and in some cows, severe pathological changes that affect reticular structure and function preclude placement of a magnet into the reticulum altogether.

It was surprising that only 53% of the foreign bodies were attached to the magnet at the time of the second radiograph even though this rate was similar to 54% obtained in an earlier study of 100 cows with TRP [18, 30]. Other authors reported much higher efficacy rates of magnets of up to 97% [9, 10, 14]. A possible explanation for this difference is that field studies involve primarily acute and often uncomplicated cases, whereas the present study involved cases that did not respond to treatment by the referring veterinarian. Furthermore, unless radiography was part of the diagnostic procedure, it can be assumed that not all cattle treated successfully with a magnet indeed had TRP and that part of the high efficacy rates may be based on misdiagnosis [30]. The efficacy of a magnet depends greatly on the position of the foreign body within the reticulum; foreign bodies lying on the ventral aspect of the reticulum or in an upright position are more likely to become attached to a magnet than foreign bodies that have no contact with the ventral aspect of the reticulum or have perforated the reticulum. In a previous study [30] magnets were most effective (92%) when foreign bodies were lying flat on the ventral aspect of the reticulum, whereas in the present study magnets were most efficacious with foreign bodies in an upright position on the ventral aspect of the reticulum (75%). The efficacy of a magnet administered orally in cattle with foreign bodies that had no contact with the ventral aspect of the reticulum (54 vs. 44%) or that had perforated the reticulum (30 vs. 32%) was similar in the present and the former study. Even though a perforating foreign body is less likely to become attached to a magnet than a non-perforating foreign body, our findings show that radiographs cannot be used to determine whether administration of a magnet will be successful. Furthermore, there was no association between the severity of perforation or the length and shape of the foreign body and the efficacy of treatment. This supports our earlier recommendation that the first line of treatment in cows with TRP is administration of a magnet [30] except when a large part or the entire foreign body is situated outside of the reticulum. The response to treatment is considered positive when the rectal temperature normalises and eating and rumination improve [31] but can also be confirmed radiographically. Ultrasonography is not a suitable tool for determining the short-term response to treatment because attachment of a foreign body to a magnet cannot be visualised and it takes several months for inflammatory lesions to heal [32].

Surgical removal of foreign bodies situated on the ventral aspect of the reticulum or perforating the reticulum was generally straightforward, and the outcome of surgery was often successful even though most cases were chronic and had severe adhesions. This indicates that delaying the surgical removal of a foreign body after a period of conservative treatment is not necessarily a disadvantage. This is also supported by the observation that most fibrinous changes involving the reticulum can regress within 6 months [32]. Foreign bodies that were entirely outside of the reticulum (n = 9) could not always be found. Before a reticular abscess is incised and drained from within the reticulum, the surgeon must confirm that the abscess is closely adhered to the reticulum.

The success rates of conservative and surgical treatment were 82 and 90%, respectively, which was similar to previously reported rates of 84 [33] and 89% [34] for conservative treatment and 90–95% [2] for surgical treatment of acute cases.

Of 299 foreign bodies retrieved surgically, 141 (47.2%) were pieces of fence wire and 121 (40.5%) were nails, confirming observations of other authors that these types of foreign bodies are most common [35–37]. Aluminium foreign bodies were rare, which was in agreement with another study [38], and wires from cut tires used to weigh down tarps covering silage were not identified [38–40], because silage in Switzerland is usually stored in upright silos rather than in bunker silos that are covered with a tarp and tyres. Non-magnetic foreign bodies have been reported to be rare [36] and we encountered only 6%. These foreign bodies are difficult to treat medically and may be diagnosed when a magnet and a foreign body are seen together in the reticulum, but there is no contact between them [41]. There also have been two reports of an increased incidence of TRP in the vicinity of small airports where adjacent hay fields were contaminated with worn wire bristles from brushes used to clean the runways [42, 43].

## Conclusions

Initial treatment for TRP should include a magnet administered orally and antibiotics. When there is no response to conservative treatment within 3–4 days, the cow is re-assessed, ideally using radiography, and the treatment options are re-evaluated. Surgery is indicated only when a foreign body that has penetrated or perforated the reticulum fails to fully attach to the magnet.
